# Heterozygous *CAV1 *frameshift mutations (MIM 601047) in patients with atypical partial lipodystrophy and hypertriglyceridemia

**DOI:** 10.1186/1476-511X-7-3

**Published:** 2008-01-31

**Authors:** Henian Cao, Lindsay Alston, Jennifer Ruschman, Robert A Hegele

**Affiliations:** 1 Vascular Biology Group, Robarts Research Institute, London, Ontario, Canada; 2 Department of Medicine, Schulich School of Medicine and Dentistry, University of Western Ontario, London, Ontario, Canada; 3 Division of Human Genetics, Cincinnati Children's Hospital Medical Center, Cincinnati, Ohio, USA

## Abstract

**Background:**

Mice with a deleted *Cav1 *gene encoding caveolin-1 develop adipocyte abnormalities and insulin resistance. From genomic DNA of patients with atypical lipodystrophy and hypertriglyceridemia who had no mutations in any known lipodystrophy gene, we used DNA sequence analysis to screen the coding regions of human *CAV1 *(MIM 601047).

**Results:**

We found a heterozygous frameshift mutation in *CAV1*, designated I134fsdelA-X137, in a female patient who had atypical partial lipodystrophy, with subcutaneous fat loss affecting the upper part of her body and face, but sparing her legs, gluteal region and visceral fat stores. She had severe type 5 hyperlipoproteinemia, with recurrent pancreatitis. In addition, she had some atypical features, including congenital cataracts and neurological findings. Her father was also heterozygous for this mutation, and had a similar pattern of fat redistribution, hypertriglyceridemia and congenital cataracts, with milder neurological involvement. An unrelated patient had a different heterozygous frameshift mutation in the *CAV1 *gene, designated -88delC. He also had a partial lipodystrophy phenotype, with subcutaneous fat loss affecting the arms, legs and gluteal region, but sparing his face, neck and visceral fat stores. He also had severe type 5 hyperlipoproteinemia, with recurrent pancreatitis; however he had no clinically apparent neurological manifestations. The mutations were absent from the genomes of 1063 healthy individuals.

**Conclusion:**

Thus, very rare *CAV1 *frameshift mutations appear to be associated with atypical lipodystrophy and hypertriglyceridemia.

## Background

Lipodystrophies are a heterogeneous group of diseases that result in abnormal fat distribution and severe insulin resistance [1-5]. The molecular basis for several forms of partial and complete lipodystrophy syndromes have been characterized [6], but some patients with inherited lipodystrophies do not have mutations in the known lipodystrophy genes. Finding other candidate genes for evaluation can be challenging because the families of such probands are often small, so positional cloning (or linkage analysis) using genome-wide marker sets cannot be performed. Another approach to find causative mutations is to select human candidate genes by analogy from genes that have been manipulated in animal models with a comparable phenotype. For instance, mice with induced deficiency in *Cav1 *encoding the cell surface protein caveolin-1 (MIM 601047) showed depleted and abnormal adipocytes with insulin resistance and severe hypertriglyceridemia [7-9]. Since no human mutations in *CAV1 *have yet been reported, we screened the genomic DNA of 60 unrelated adults with partial lipodystrophy and hypertriglyceridemia and no mutation in any known lipodystrophy gene to search for coding sequence mutations in *CAV1*.

## Results

### Demographics of study sample

From a tertiary referral lipid clinic, we evaluated 60 patients (80% female) who had partial lipodystrophy, with diabetes or moderate to severe (type 4 or 5) hypertriglyceridemia. Age, body mass index, untreated fasting plasma cholesterol and triglycerides (mean+standard deviation [SD]) were, respectively, 52.5 ± 14.0 years, 26.2 ± 3.8 kg/m^2^, 6.9 ± 5.0 mmol/L and 11.1+10.0 mmol/L. All subjects consented to DNA analysis. No coding sequence mutations were found in *LMNA*, *PPARG*, *BSCL2 *or *AGPAT2 *genes encoding nuclear lamin A/C, peroxisome proliferator-activated receptor gamma, seipin or 1-acyl-sn-glycerol-3-phosphate acetyltransferase, respectively.

### Characterization of patients with *CAV1 *mutations

**Figure 1:**
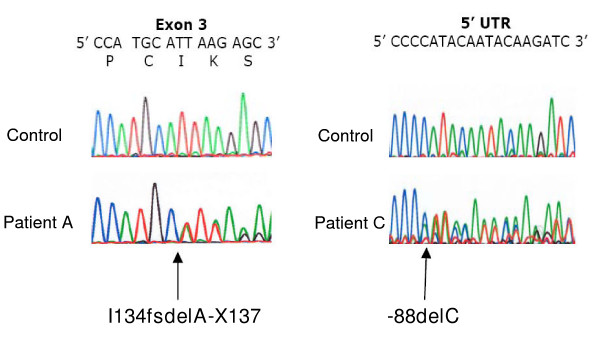
Electropherogram scans showing the novel heterozygous *CAV1 *frameshift mutations in the lipodystrophy patients. The left half of the figure shows a portion of *CAV1 *exon 2 from genomic DNA of a control subject and Patient A. The right half of the figure shows a portion of *CAV1 *5' untranslated region (5'UTR) from genomic DNA of a control subject and Patient C. For each tracing, normal nucleotide sequence is shown in the top line of letters, with single letter amino acid codes and codon numbers beneath for exon sequence. The position of each inserted nucleotide is indicated by the arrows for the respective mutations I134fsdelA-X137 and -88delC.

Two rare heterozygous coding sequence variants in *CAV1 *were found among the screened patients, I134fsdelA-X137 in one patient and -88delC in another (Figure 1). These frameshift mutations were absent from the genomes of 1063 normolipidemic control subjects. The I134fsdelA-X137 mutation predicted premature termination, with loss of the carboxy terminal half of the protein product. This mutation was subsequently found in the proband's father, who had a similar phenotype. The -88delC mutation occurred within the 5'-untranslated region with a potential effect on the reading frame. The clinical features of the father and daughter with the heterozygous I134fsdelA-X137 mutation and the singleton patient with the heterozygous -88delC mutation are summarized in Table 1.

**Table 1:** Clinical and biochemical features of atypical lipodystrophy patients with *CAV1 *mutations

**Attribute**	**Patient A**	**Patient B**	**Patient C**
Ancestry	Northern Europe	Northern Europe	Northern Europe
*CAV1 *mutation name	I134fsdelA-X137	I134fsdelA-X137	-88delC
age at assessment (years)	28	55	35
sex	female	male	male
lipodystrophy onset	birth	birth	adulthood
BMI (kg/m^2^)	25.0	24.5	25.6
waist circumference (cm)	86	88	92
Subcutaneous fat changes:			
face	decrease	decrease	increase
arms	decrease	decrease	decrease
gluteal region	increase	increase	decrease
thighs	increase	increase	decrease
calves	increase	increase	decrease
visceral	increase	increase	increase
			
age of diabetes onset (years)	none	30	33
hypertension and age of onset	none	none	33
hyperlipoproteinemia	type 5	type 4	type 5
pancreatitis	recurrent	no	recurrent
highest historical plasma triglyceride	20.4 mmol/L	80 mmol/L	16.5 mmol/L
acanthosis nigricans	present	absent	present
			
congenital cataracts	bilateral	bilateral	absent
atypical retinitis pigmentosa	bilateral	bilateral	absent
			
tinnitus	moderate	absent	absent
			
nystagmus	severe	absent	absent
spastic ataxia	severe	mild	absent
dysdiadochokinesia	severe	mild	absent
muscle power in lower limbs	diminished (4/5)	normal	normal
sensory glove/stocking neuropathy	severe	mild	absent
Babinski sign	bilateral	absent	absent
finger past-pointing	severe	absent	absent
			
other comments	wheel-chair bound since age 20; similarly affected paternal aunt (deceased)	Patient A's father; legally blind	requires 120 U of insulin daily

The history and clinical features of the I134fsdelA-X137 mutation proband (Patient A) and her affected father (Patient B) have been previously reported [10]. Briefly, the proband, who was assessed at age 28 years, was a woman of Northern European origin who was wheelchair bound since early adulthood. At birth, she was noted to have a lack of facial and upper body subcutaneous fat, micrognathia and congenital cataracts. Later, diplopia, tinnitus and a central auditory processing defect were noted. She had severe type 5 hyperlipoproteinemia with recurrent pancreatitis; she had insulin resistance but no diabetes. On examination, she was noted to have orthostatic hypotension, a high arched palate, taut skin with sparing of subcutaneous fat on buttocks, hips and thighs. She was noted also to have webbed toes, acanthosis nigricans, retinitis pigmentosa, iris deposits, marked nystagmus, ocular dysmetria, past-pointing on finger-nose testing, dysdiadochokinesis, a spastic-ataxic gait, spontaneous clonus of legs, bilateral Babinski signs, reduced power in the lower extremity and lost vibration sense in a glove-and-stocking distribution. She had leg spasms, a stiff gait, paresthesiae, imbalance and urinary incontinence. Investigations showed lipemic plasma with markedly elevated serum cholesterol (9.3 mmol/L) and triglycerides (20.4 mmol/L). Her father (Patient B), a 55 year old diabetic man of Northern European origin, had similar body fat distribution to his daughter. He also had congenital cataracts and retinitis pigmentosa, but no neurological complaints, and was ambulatory and well-functioning. On examination, he was noted to have absent facial, neck, and limb fat with increased abdominal and lower back subcutaneous fat. He also had decreased sensation in both legs, with normal muscle power and tone; an unsteady narrow-based gait was elicited. He had milder biochemical abnormalities than his daughter, with mild hypertriglyceridemia and no history of pancreatitis. Of note, Patient B's sister (the paternal aunt of Patient A) died from an undefined neurological condition at age 40. Medical records showed a clinical presentation similar to Patient A, with analogous fat redistribution, leg weakness and ataxia beginning at age 18 and requirement for a wheelchair at age 20 [10]. Patient C, a heterozygote for the *CAV1 *-88delC mutation, was 35 years old at assessment. He was noted in his early 20's to have redistributed fat stores, with a relative loss of subcutaneous fat on his arms, gluteal region, thighs and calves and increased visceral fat on abdominal ultrasound examination. He had severe hypertriglyceridemia (range 10 to 80 mmol/L) beginning in his late 20's, with recurrent episodes of pancreatitis. He was diagnosed with diabetes at age 33 and currently takes 120 units of insulin daily in addition to fenofibrate 200 mg daily. Physical examination revealed fat redistribution, with no ocular, neurological, dermatologic or musculoskeletal findings.

## Discussion

Caveolin-1 is expressed in numerous tissues and is a central component of cell surface caveoli, which are plasma membrane microdomains that regulate signaling pathways and processes such as cell migration, polarization, proliferation and especially endocytosis [11-13]. *Cav1 *appears to have a significant role in murine adipocyte metabolism, and disruption of the gene leads to severe hypertriglyceridemia, insulin resistance and adipocyte abnormalities, which are all features that are consistent with human partial lipodystrophy [7-9]. Our genetic findings suggest that *CAV1 *might also have a role in human adipose, insulin and triglyceride metabolism, albeit only two frameshift mutations in *CAV1 *were found among 60 lipodystrophic patients with a normal coding sequence of known lipodystrophy genes.

The frameshift mutations would be expected to result in a compromised gene product from the affected allele. The I134fsdelA-X137 mutation co-segregated with the phenotype from father to daughter, but the more severe phenotype in the daughter suggests that other factors – perhaps genetic or non-genetic – can modulate the severity of the phenotype. The presence of a comparably severe phenotype in Patient A's paternal aunt suggests that gender might also modulate the severity of phenotypic expression in carriers. Also, the variable organ system involvement – the ocular and central nervous systems in Patients A and B but not in Patient C – is consistent with the variable multi-system phenotypes seen in *Cav1 *deficient mice depending on genetic background [11-13]. However, heterozygous *Cav1 *deficient mice do not show any phenotype, again suggesting that other factors are required for phenotypic expression in these patients. Unfortunately, we could not further extend the families in order to document phenotypes seen in other *CAV1 *mutation carriers at present, although this is planned for the future.

## Conclusion

The association of these rare frameshift mutations in *CAV1 *with atypical presentations of partial lipodystrophy with insulin resistance and hypertriglyceridemia, the absence of these mutations from healthy controls together with evidence from mouse models link the *CAV1 *gene with human metabolic phenotypes. Since only two mutations were found among 60 screened subjects, *CAV1 *mutations are a very rare cause of lipodystrophy and hypertriglyceridemia.

## Methods

### Genomic DNA analysis

The exons and intron-exon boundaries of *CAV1 *were amplified and bi-directionally sequenced using the primers and conditions in Table 2. All coding sequence mutations were confirmed by a second sequencing reaction performed on another day. The frequency of the *CAV1 *exon 3 I134fsdelA-X137 variant was determined in healthy control subjects using genomic DNA amplification with primers 5'-ATG GAT ACT GAA TAG TGG GTT TTT and 5'-TGT TGC TGT ATT AGC AAC TTG GA. The 509 bp product was digested with restriction enzyme *Nsi*I (New England Biolabs, Mississauga, ON) and fragments were resolved on 2% agarose gels. The digested fragments of the wild-type allele were 280 and 229 base pairs (bp) in size, while the digested fragment of the mutant allele was 509 bp in size. The frequency of the *CAV1 *exon 1 -88delC variant was determined in healthy control subjects using genomic DNA amplification with primers 5'-AG ATG ATG CAC TGG GAA AA and 5'-CTA GGC CCC CTC TCC ATT AG, with allele specific diagnostic (SNaPShot) primer 5'-GAT CTT GTA TTG TAT GGG GG.

**Table 2:** Primers used for sequencing *CAV1*

Exon	Primers	Fragment size (nucleotide base pairs)
1	F-5' GAG ATG ATG CAC TGG GAA AA R-5' CTA GGC CCC CTC TCC ATT AG	633
2	F-5' GTA GCT GTC GGA GCG GTT AG R-5' GGA GCT CCC ACA CAT CAA AC	480
3	F-5' ATG GAT ACT GAA TAG TGG GTT TTT R-5' TGT TGC TGT ATT AGC AAC TTG GA	509

## Competing interests

The author(s) declare that they have no competing interests.

## Authors' contributions

HC and LA carried out all molecular analysis and participated in manuscript writing. JR participated in obtaining patient samples for analysis. RAH conceived of the study, participated in its design, analysis, interpretation and manuscript preparation. All authors approved the final version of the manuscript.
